# Assessing the subjective quality of smartphone anterior segment photography: a non-inferiority study

**DOI:** 10.1007/s10792-022-02437-9

**Published:** 2022-08-26

**Authors:** Raghav Goel, Carmelo Macri, Bobak Bahrami, Robert Casson, Weng Onn Chan

**Affiliations:** 1 Discipline of Ophthalmology and Visual Sciences, The University of Adelaide, North Terrace, Adelaide, South Australia 5000 Australia; 2 Department of Ophthalmology, Royal Adelaide Hospital, Port Road, Adelaide, South Australia 5000 Australia

**Keywords:** Anterior segment, Smartphone photography, In-built slit lamp camera, Non-inferiority, Telehealth

## Abstract

**Purpose:**

To assess the subjective quality of anterior segment photos taken from a smartphone camera adapted to the slit lamp compared to a commercial inbuilt slit-lamp camera.

**Methods:**

Non-inferiority study. Five paired images of the anterior segment of normal eyes were taken using an iPhone 11 (Apple, Inc., Calif., USA) camera attached to a universal slit-lamp adaptor and a commercial inbuilt slit-lamp camera (Haag-Streit Diagnostics, Bern, Switzerland). Images were collated into a survey in which ophthalmology students, residents, registrars, and consultants participated to select the image taken from the inbuilt slit-lamp camera. If the image quality was subjectively indistinguishable, we expected a 50:50 split for each photograph that was presented. We selected a 10% non-inferiority margin, with the hypothesis that no less than 40% of images believed to be from the conventional camera were in fact from the smartphone camera.

**Results:**

There were 27 respondents in the survey: ophthalmology consultants (*n* = 7), registrars (*n* = 10), residents (*n* = 7), intern (*n* = 1) and students (*n* = 2). The mean correct identification across the respondents was 11.3 out of 25 (45.2%) images. Overall, the smartphone camera was non-inferior to the inbuilt slit-lamp camera (*p* < 0.001). The non-inferiority of the smartphone camera was significant for consultants (47.4%, *p* < 0.01), registrars (47.6%, *p* < 0.001) and residents (37.7%, *p* < 0.0001).

**Conclusions:**

Anterior segment images obtained with a smartphone camera were non-inferior to the commercial inbuilt slit-lamp camera. Smartphone cameras may be a non-inferior tool for communication of anterior segment images having implications for the ease of access to quality telehealth consultations.

**Supplementary Information:**

The online version contains supplementary material available at 10.1007/s10792-022-02437-9.

## Introduction

In Australia, approximately 1.4% of all emergency department (ED) presentations are related to eye disorders [2]. Anterior segment pathology accounts for more than 90% of ED presentations related to eye disorders [3]. Specialist consultation is frequent given medical officers report decreasing confidence in slit lamp usage and management of ocular emergencies in the ED [4].

Ophthalmology consults may be performed virtually with the use of high-quality photographs, increasingly relevant with the increased uptake of telemedicine in response to the COVID-19 pandemic [5, 6]. Inbuilt slit-lamp cameras are an effective means for anterior segment photography. Despite a large proportion of EDs having access to slit lamps, medical officers report a lack of training to use them [7]. In addition, rural EDs are less likely to have access to slit lamps [8].

Conversely, smartphones are widely available and offer a wide range of functionalities. The increasing camera quality of smartphones, low cost and universality have sparked interest in their utility in ophthalmology [9]. Slit lamp photography is possible with the use of inexpensive smartphone adaptors that offer comparable image quality to inbuilt slit-lamp cameras. Observations made by previous investigators have demonstrated smartphone cameras offering good image quality, however, reproducibility and agreement on this are lacking [10–13].

Given the ease of access to a smartphone camera, there may be a role in using them to image anterior segment pathology in primary and tertiary care centres. This can be used for consultation purposes without the need for specialised slit-lamp cameras. This pilot study was designed to assess whether ophthalmology staff can discriminate between images taken of the anterior segment in healthy eyes from an inbuilt slit-lamp microscope camera compared to that of a smartphone camera attached to a universal slit-lamp adaptor.

## Methods

**Fig. 1:**
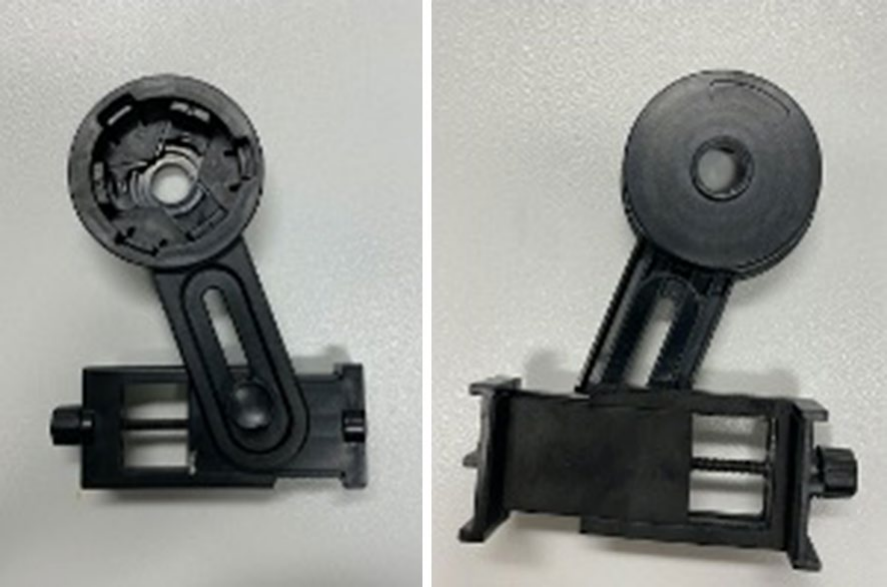
Adjustable slit-lamp adaptor used to attach the smartphone to the slit lamp eyepiece

**Fig. 2:**
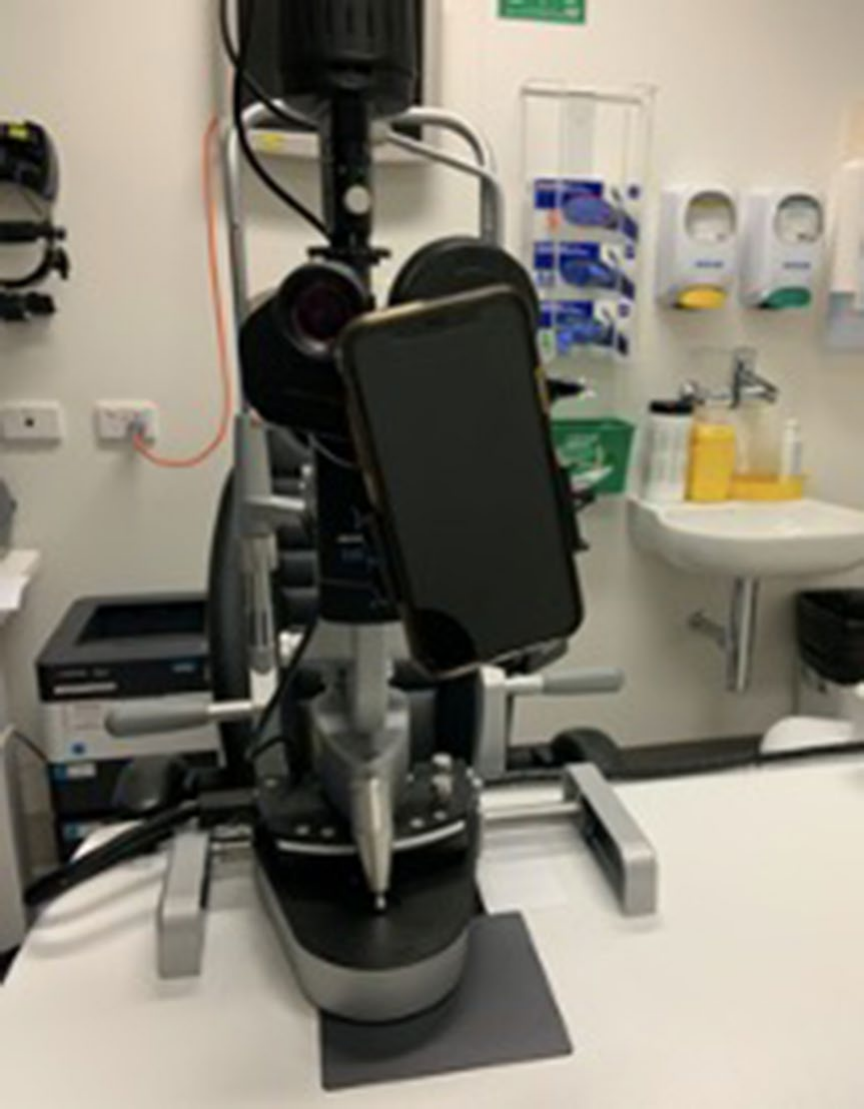
Set-up of the slit lamp adaptor on the Haag-Streit BQ-900 slit-lamp using an iPhone 11 (Apple, Inc., Cupertino, Calif., USA)

All participants were recruited from the Royal Adelaide Hospital (RAH) Ophthalmology Department. Ethics approval was acquired from the local Human Research Ethics Committee at the Central Adelaide Local Health Network Research office (reference 13716). Consent was obtained from five staff volunteers in the department to acquire images of their anterior segment. An inexpensive adjustable slit-lamp phone adaptor was utilised as depicted in Fig. 1 [14]. The iPhone 11 (Apple, Inc., Cupertino, Calif., USA) was mounted onto the adaptor and placed over the right eyepiece of the slit lamp (Fig. 2). At the time, iOS™ (14.3) software was installed on the iPhone 11.

**Fig. 3:**
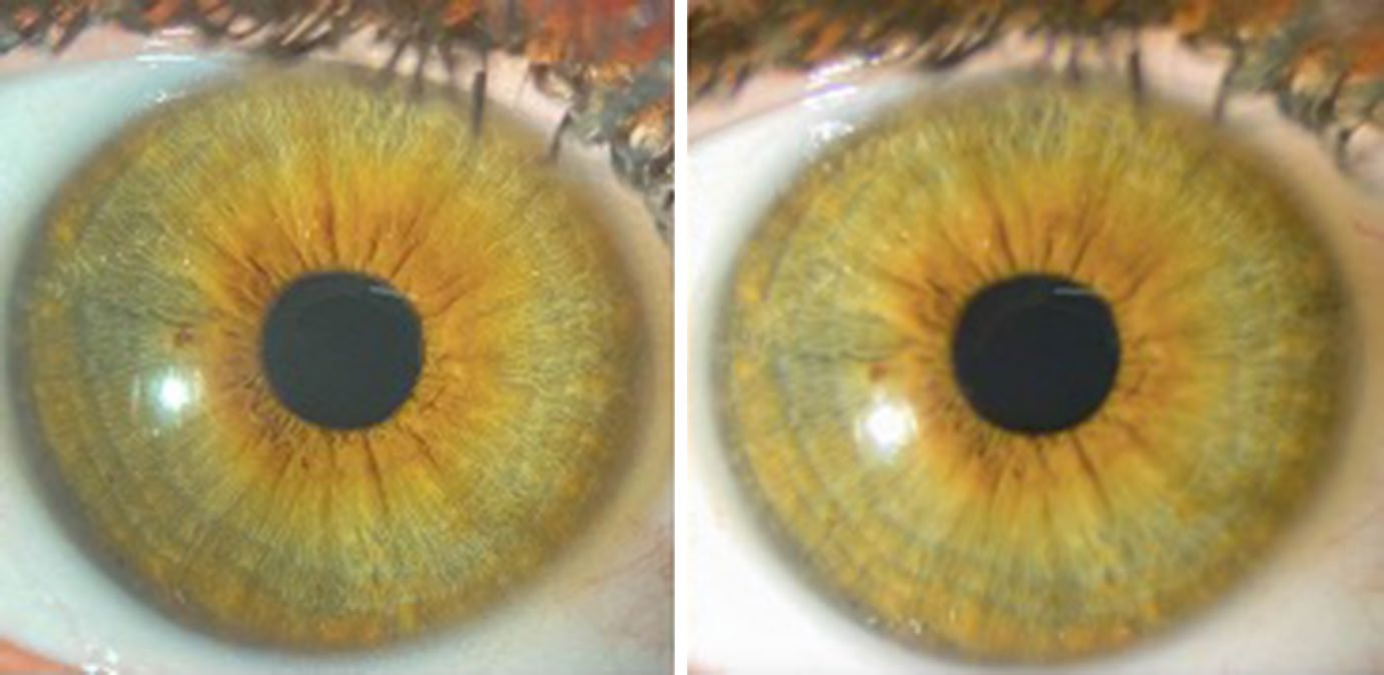
Diffuse Illumination example photo of a participant. Left: image taken using iPhone 11. Right: image taken using Image module IM-900 of the slit lamp

**Fig. 4:**
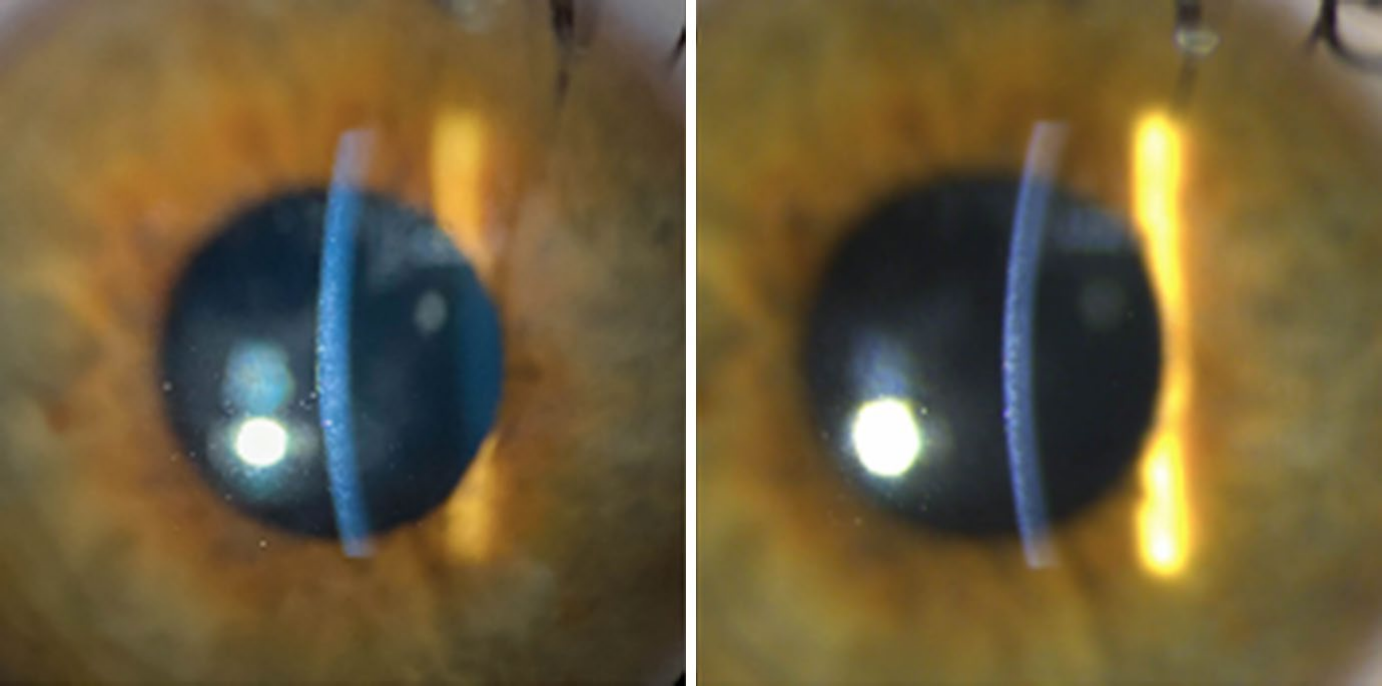
Corneal slit photographs were taken by the iPhone 11 (left) and the built-in slit lamp camera (right)

A survey with each pair of images for all subjects was created using Google Forms (Alphabet Inc., California, USA). An invitation link to the survey was provided to medical students, interns, ophthalmology residents (i.e., unaccredited trainees), registrars (i.e., accredited trainees), consultant ophthalmologists. Participants were given the option of answering anonymously and recording their level of expertise. The survey consisted of 25 comparison questions and was blinded to individuals who were not involved in the imaging process. The questions asked the participants to select image captured by the commercial slit-lamp camera (Figs. 3 and 4). The correct responses were recorded.

Survey design sample size calculations allowing for correlated data indicated that approximately 650 data points would be required to detect a 10% margin in non-inferiority where the null hypothesis assumed a 50:50 split with 80% power and alpha at 0.05. The p-values were calculated with a z-test and the correct variance estimate on the proportion was calculated by modelling the study as a clustered design using the *survey* package in R (version 4.0) with the individual as the unit of a cluster [15, 16]. A Bonferroni correction was applied for sub-group analyses.

The slit-lamp images were taken using the integrated Haag-Streit IM900 Imaging module with Eyesuite™ software (Haag-Streit Diagnostics, Bern, Switzerland) [17]. The same iPhone and slit lamp were used throughout the study. Two medical officers (MO) took sets of three photographs using both cameras, for five separate imaging techniques. These techniques were diffuse illumination, narrow corneal slit, sclerotic scatter, lens optical section, and iris retro-illumination (see Appendix, Table 1s@@). The anterior segment photos were captured simultaneously using the two cameras where one MO captured photos using the smartphone on the slit lamp, whilst the other using the Eyesuite™ software on the computer.

The settings enabled on the iPhone camera included ‘high dynamic range (HDR)’ and ‘autofocus’. The slit lamp settings were adjusted based on the imaging technique but were kept constant for all the subjects (see Appendix, Table 1s@@). The best quality images were chosen from a set of three, to simulate out-of-study conditions in which the best photos of multiple would be selected. The pairs of photos were cropped using Adobe Lightroom™ 4.1.1 (Adobe, San Jose, California, USA) to display similar structures in each photo. The cropped images were then light adjusted using Lightroom’s *Auto* function, in conjunction with adjusting the *Exposure* slider, to display visibly equal exposure. This was done to ensure survey participants would only use photo quality to discriminate the image pairs and prevent different light exposure or field angles to influence the result. The time taken for this was approximately a minute for each set of photos.

## Results

**Fig. 5:**
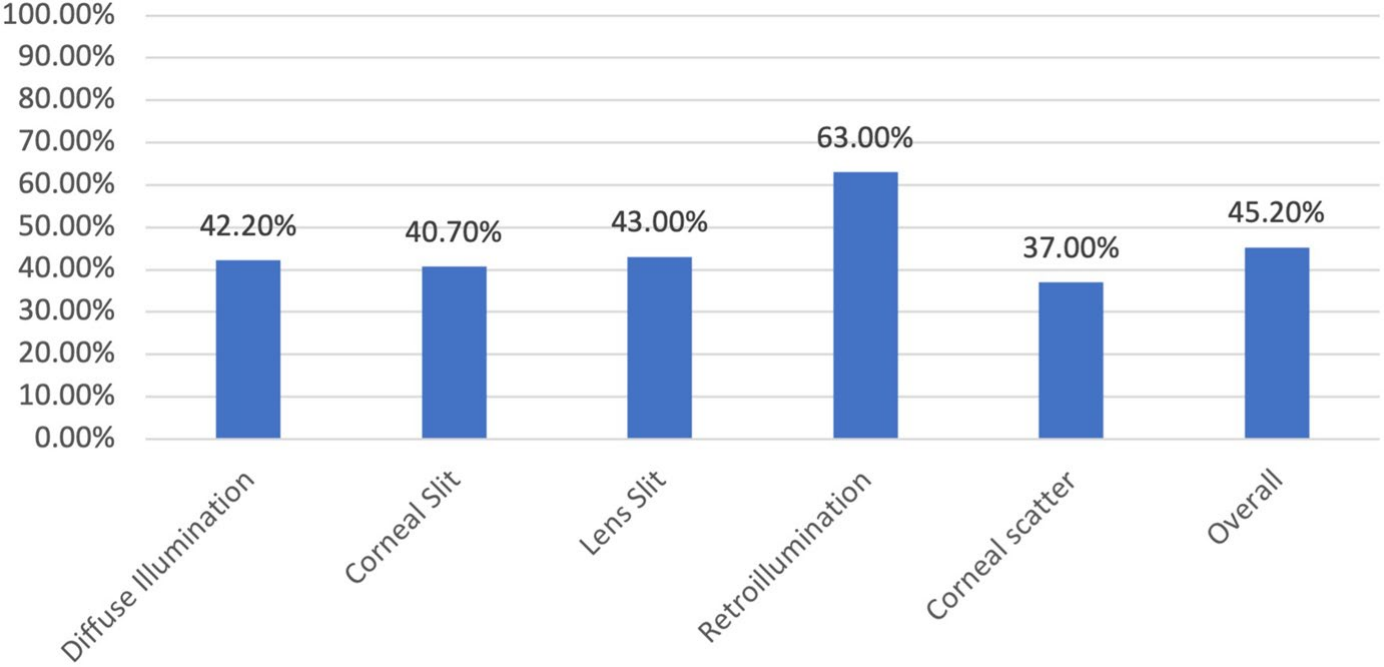
Mean score for each type of image. Note: All survey respondents were used for this calculation. No cohort was omitted

Twenty-seven respondents completed the survey comprising twenty-five image questions and two quality control questions. There was a total of 675 data points extracted from the survey. Overall, the respondents achieved a mean correct score of 11.3 out of 25 (45.2%, SD 14.0) in the survey for correctly identifying the inbuilt slit lamp photos. Thus, 54.8% of respondents believed the smartphone camera was the commercial slit-lamp camera image, signifying that the smartphone camera is non-inferior to the inbuilt slit-lamp camera (*z*-test, *p*-value < 0.001). The mean score for each subset of images is shown in Fig. 5.

The survey also noted the category of the respondent as a consultant, registrar, resident, intern or student. The survey was mostly completed by the consultants 25.9% (*n* = 7), registrars 37% (*n* = 10) and residents 25.9% (*n* = 7). The other respondents were interns 3.7% (*n* = 1) and students 7.4% (*n* = 2). The consultant group were accurate 47.4% of the time incorrectly identifying slit-lamp camera image (*p*-value < 0.01). Similarly, registrars had a correct guess rate of 47.6% for slit-lamp images (*p*-value < 0.001) of the time and the residents strongly favoured the smartphone camera with only 37.7% correct guess rate for slit lamp images (*p*-value < 0.0001). The correct guess rate for slit-lamp photos is depicted in Table 1.

**Table 1:** Mean scores of the respondents for correctly identifying the inbuilt slit-lamp image and non-inferiority significance level

Seniority	Respondents	Mean (%)	Mean score (SD)	95% confidence interval	P-Value
Consultant	7	47.4%	11.9 (3.5)	11.8–12.0	P = 0.0085
Registrar	10	47.6%	11.9 (3.1)	11.8–12.0	P < 0.001
Resident	7	37.7%	9.4 (3.7)	9.3–9.5	P < 0.0001
Overall	27	45.2%	11.3 (3.4)	11.25–11.35	P < 0.0001

The data for intern (n = 2) and students (n = 1) was omitted due to insufficient sample size

**Fig. 6:**
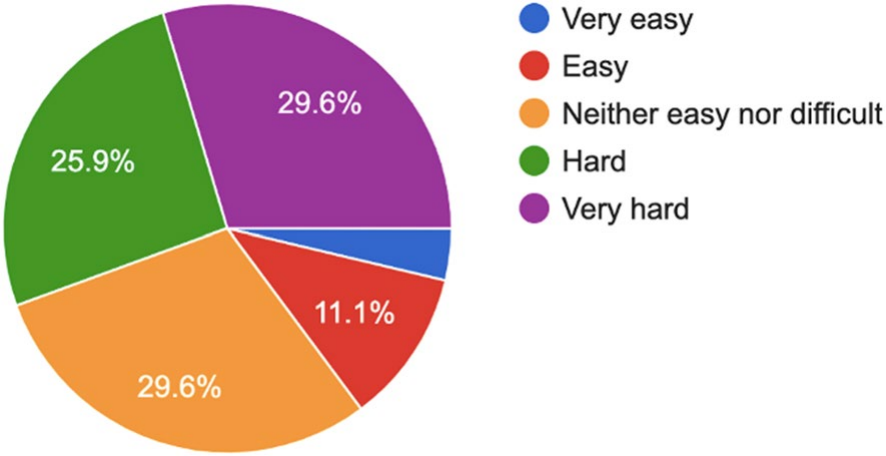
Difficulty reported by participants in differentiating between the images

Respondents were asked if the images were of comparable quality and 20 out of 27 respondents (74.1%) believed they were. The respondents who did not agree with this finding achieved a mean score of 9.3 out of 25 (*n* = 7, 37.2%), compared to those that agreed had a mean score of 12 (*n* = 20, 48%) for correctly identifying slit lamp photos. Respondents also reported the difficulty experienced in comparing images as per Fig. 6.

## Discussion

In this study conducted by medical personnel with varying ophthalmic expertise, the smartphone camera was non-inferior to the commercial inbuilt slit-lamp camera. This suggests that adequate anterior segment imaging can be obtained without specialist equipment, offering more accessible telehealth opportunities for consultation and specialist opinion. This may reduce the time for specialist involvement in patient care and provide cost savings by reducing the need for expensive photographic equipment.

The pandemic era paves the way for smartphone devices to be used as a valuable tool for telehealth consultations in ophthalmology. Uses range from a tool to detect ptosis, cataracts, measurement of toric alignment, evaluation of globe anatomy and trachoma surveillance [18–23]. Previous pilot studies have shown that anterior segment photos taken through smartphone cameras such as the iPhone 4 s and 5 s were all of poorer quality when compared to the Zeiss photo slit lamp camera (Carl Zeiss Meditec, Dublin, Calif., USA) [24]. Others have studied anterior segment images post-cataract operation or with the use of different smartphone attachments such as the 90-dioptre lens [25–29]. None of these studies have compared if the subjective quality of anterior segment images captured by a smartphone camera and an inbuilt slit-lamp camera.

In contrast to the above studies, this study shows that anterior segment images taken from iPhone 11 are non-inferior to the Haag Streit BQ-900 imaging module as perceived by the ophthalmology staff. Interestingly, the resident group tended to strongly favour the smartphone images, as they correctly identified the slit lamp photos only 37.7% of the time. This preference of the resident group may be due to their education level, age, and smartphone usage which can cause deviation in the results.

The reasons for non-inferiority are multifactorial. The 12-megapixel camera on the iPhone 11 is comparable to the high sensitivity and a wide dynamic range provided by the Haag Streit Imaging Module (IM) 900. The resolution offered by iPhone 11 camera (4032-by-3024 pixels) is superior to the Haag-Streit IM 900 module (1920-by-1200 pixels) [17, 30, 31]. The other key differences are the software and functionalities of the two cameras. Similar to other smartphones, the iPhone 11 offers features such as auto-focus and optical image stabilisation capabilities that the IM 900 module does not. However, it can be argued that the IM 900 has superior stabilisation abilities as it is integrated into the slit lamp as compared to the smartphone attached to an adaptor which requires adjustments. Furthermore, the software of the iPhone 11 obtains up to 10 updates every year as compared to just an annual update for the IM 900 module which may enhance its camera’s performance [32, 33].

This study shows that the combination of a slit-lamp adaptor and smartphone has its advantages. Firstly, the adaptors are inexpensive, such as the one used in this pilot study (AUD $20) as compared to inbuilt slit-lamp cameras which costs upwards of AUD $20,000 (excluding costs of ongoing maintenance) [14, 34]. Secondly, the slit lamp illumination system provides clear image quality at higher magnification due to the reduced focusing distance to the eye [35]. The smartphone uses the slit lamp’s illumination at magnification to capture sharper images as compared to using the zoom function on the smartphone alone. No additional light source from the smartphone also results in decreased reflection on the ocular surface [36]. Lastly, the adaptors can be attached to most smartphones, are transportable and suitable for most slit lamps.

Applications of images captured from this setup are numerous. Sink, Blatt, Yoo et al. compared diagnosis achieved through remote smartphone photographs to those of in-office exams for common ophthalmology presentations such as external eye diseases or red-eye pathology. The results showed that the remote and in-office diagnosis made by different specialists were in agreement 93% of the time [37]. This pilot study supports the use of anterior segment images acquired with a smartphone camera to diagnose anterior segment pathology as they are similar to the inbuilt slit lamp camera. The inbuilt slit lamp camera is usually the benchmark for taking ocular photos and as the ophthalmology staff are unable to distinguish between the two cameras, they will likely find the smartphone images to be sufficient to make a diagnosis. Furthermore, new smartphone cameras providing stereoscopic images allow for tele-ophthalmology viewers to see in 3D similar to a slit lamp, furthering the potential applicability of these mounts [38, 39]. This can also be integrated with machine learning in the future to assess for anterior chamber depth and grading of cataracts which has potential generalizability to the primary care setting [39, 40].

There are some issues with the two imaging systems that can hinder the quality of photographs. The inbuilt slit-lamp cameras are often subject to availability and are only present in well-established ophthalmology clinics. They require integrated storage space on local computers and images are not as portable. Secondly, in our experience we found a small delay between pressing the capture button to when the image is acquired. This may interfere with the quality of the image due to patient movement. Conversely, the adaptor has inherent issues such as difficulty in attachment to the rubber ring on the eyepiece. If this occurs, an alternative is to place double sided tape on the adapter to allow for better fixation. Moreover, the depth of eye piece fixation ring is often shallow and may not always bear the weight of smartphone. Thus, removal of any protective cases or accessories on the phone may allow the adapter to hold the weight of the smartphone. Also, we found that having one hand on the smartphone and other on the slit lamp for manoeuvring provided stability in capturing photos. The quality of photos may differ between each doctor using the adapter, however, with basic slit lamp examination skills it should still produce good quality photos with a short learning curve.

The limitations of the study are that it does not apply to fundus photographs captured by a smartphone camera as others have previously compared [41]. The sample size of the study was dependent on the limited number of ophthalmology staff in the department. Only ophthalmology staff were used in the survey due to their more experience and knowledge of the slit lamp and ocular anatomical structures. However, this study could be replicated at a larger scale including other disciplines in the future. Lastly, the software used for light-adjusting photos was not completely automated and subject to user bias.

## Conclusion

This pilot study demonstrates that photos captured by a smartphone camera are non-inferior to those taken with an inbuilt slit lamp camera as discriminated by medical staff in our Ophthalmology department. Anterior segment images can be captured by clinicians with the use of an inexpensive slit lamp adaptor in the absence of inbuilt photography modules. This increases the accessibility of tele-ophthalmic consultation for expert opinion in settings without proximate ophthalmologists or specialised ophthalmic imaging equipment.

## Supplementary Information

Below is the link to the electronic supplementary material.

Supplementary file1 (DOCX 14 kb)

## Data Availability

Able to easily access data on the surveys through Google Forms.
