# Supplementary material for: Assessing the subjective quality of smartphone anterior segment photography: a non-inferiority study
Source: Int Ophthalmol. 2022 Aug 26;43(2):403–10. doi: 10.1007/s10792-022-02437-9 (PMC9411845; doi:10.1007/s10792-022-02437-9)
Supplement: Supplementary file 1 — Supplementary file1 (DOCX 14 kb) [file 10792_2022_2437_MOESM1_ESM.docx]

**Appendix- Supplementary Tables**

| **Table 1s: Imaging Technique Settings on the Slit Lamp** | | | | | | |
| --- | --- | --- | --- | --- | --- | --- |
| **Image technique** | **Diffuse Illumination level** | **Slit illumination level** | **Type of illumination** | **Magnification** | **Angle of illumination arm (degrees)** | **Other** |
| Diffuse illumination | 10/10 | Not applicable | Diffuse only | 10 | 45 |  |
| Narrow corneal slit | 8/10 | 10/10 | Diffuse and slit | 25 | 30 | Slit positioned over the pupil |
| Sclerotic scatter | 9/10 | 10/10 | Diffuse and slit | 10 | 30 | Slit focused over the limbus on the cornea |
| Lens optical section | off | 10/10 | Slit only | 25 | 30 | Slit positioned over the pupil |
| Iris retro illumination | off | 10/10 | Slit only | 16 | 0 |  |
